# Supplementary figures and images for: Characterization of bidirectional gene pairs in The Cancer Genome Atlas (TCGA) dataset
Source: PeerJ. 2019 Jun 17;7:e7107. doi: 10.7717/peerj.7107 (PMC6585903; doi:10.7717/peerj.7107)

Figure S1

A

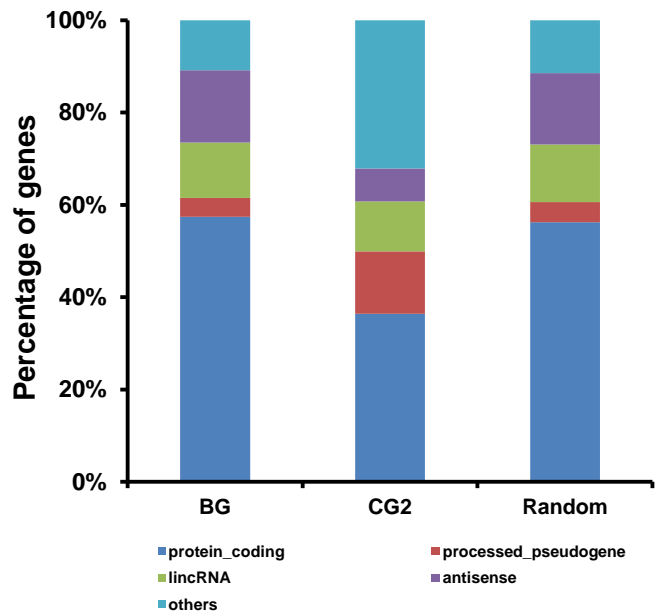

B

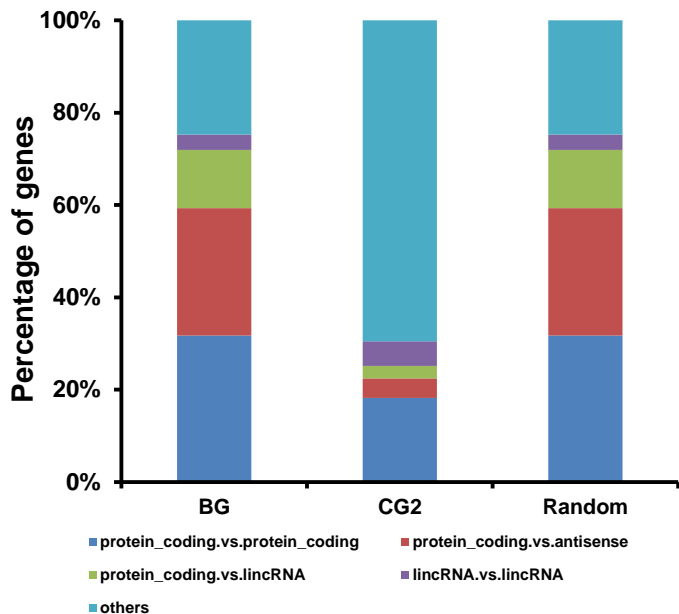

C

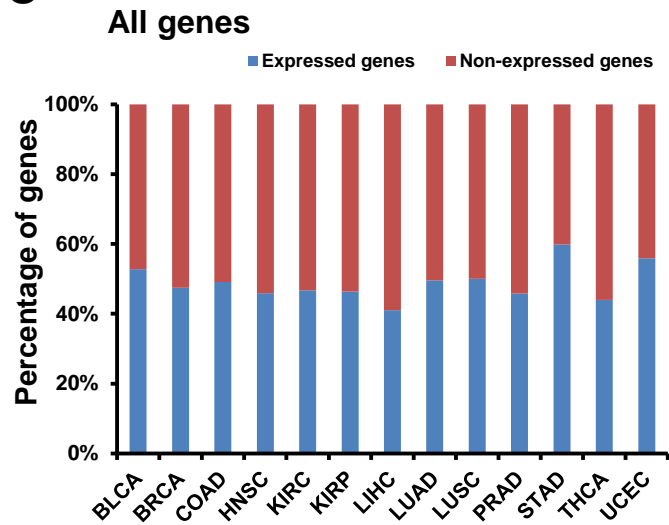

D

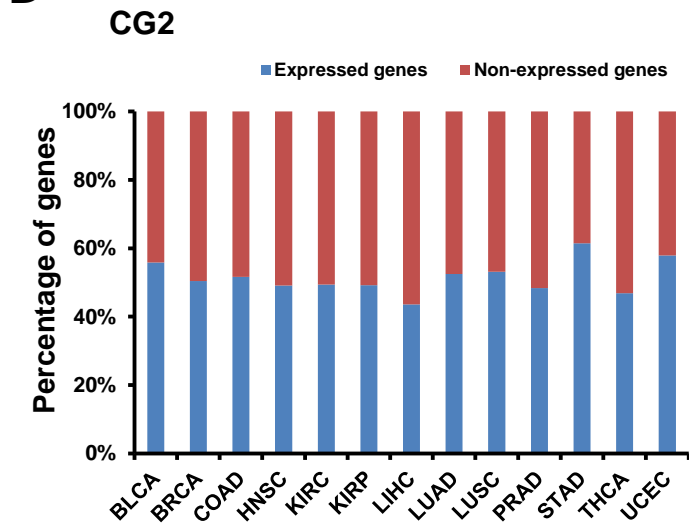

E

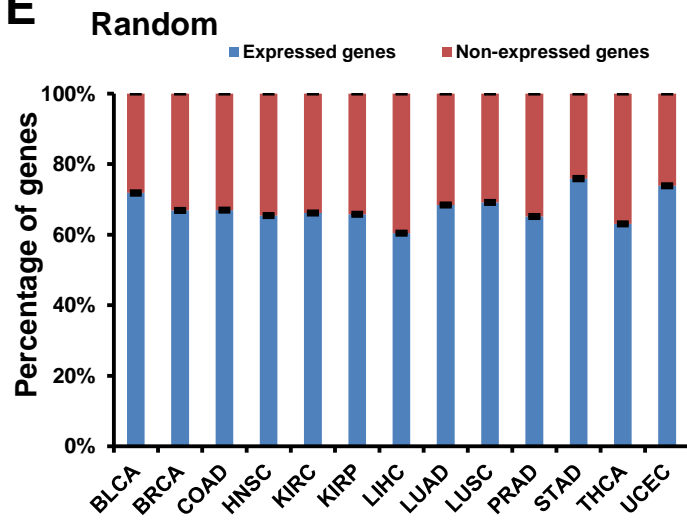

Supplement: Supplemental Information 1 — A. Distribution of gene types in bidirectional, control (CG2) and random genes. B. Distribution of the combinations of gene types in bidirectional, control (CG2) and random gene pairs. C. Bar plot showing the percentage of expressed and non-expressed genes in all human genes among TCGA datasets. D. Bar plot showing the percentage of expressed and non-expressed genes in control genes (CG2) among TCGA datasets. E. Bar plot showing the percentage of expressed and non-expressed genes in random genes among TCGA datasets. The percentages in random genes represent mean of 100 random sets and the error bars represent the standard deviation. The mean number of expressed and non-expressed genes in 100 sets of random gene pairs is used to compute significance when comparing with bidirectional genes. [file peerj-07-7107-s001.pdf]

Figure S2

A

Up-regulation

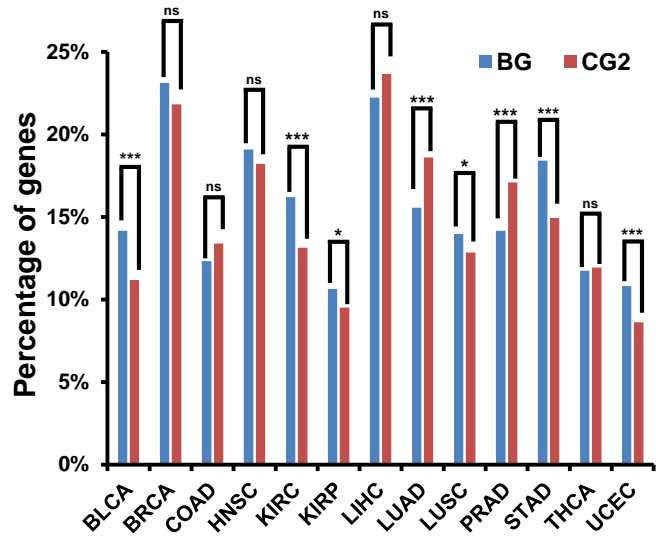

B

Down-regulation

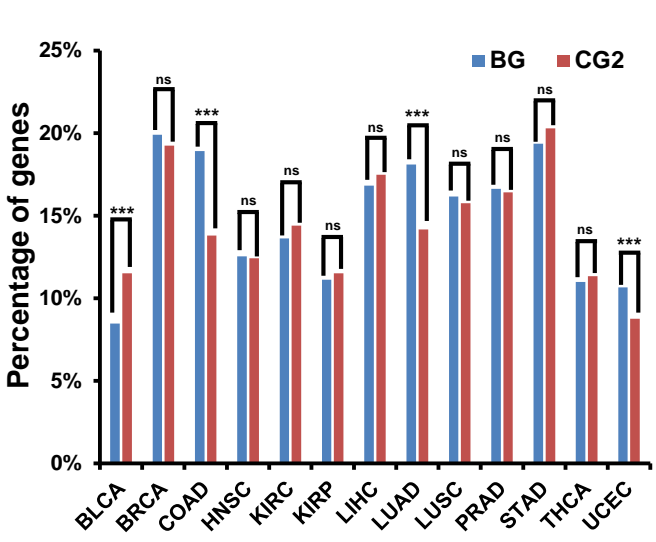

C

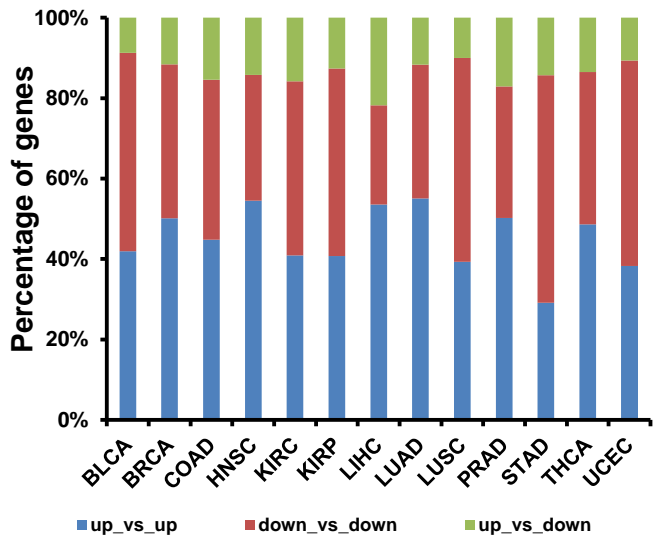

D

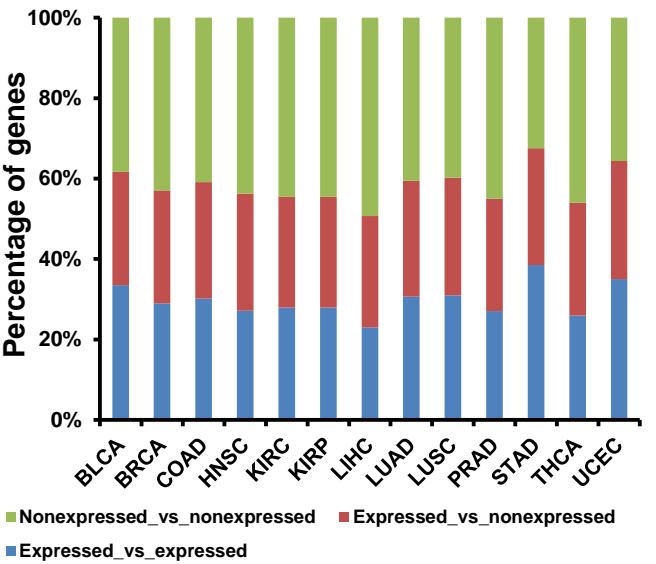

Supplement: Supplemental Information 2 — A. Percentage of up-regulated bidirectional and control genes (CG2) among TCGA datasets. X-axis, TCGA datasets. The p-values were computed by “chisq.test” function in R. “ns” indicating not significant. “*” indicating p-value < 0.05. “**” indicating p-value < 0.01. “***” indicating p-value < 0.001. B. Percentage of down-regulated bidirectional and control genes (CG2) among TCGA datasets. X-axis, TCGA datasets. The p-values were computed by “chisq.test” function in R. “ns” indicating not significant. “*” indicating p-value < 0.05. “**” indicating p-value < 0.01. “***” indicating p-value < 0.001. C. Percentage of the patterns of differentially expressed combination in control gene pairs (CG2). X-axis, TCGA datasets. D. Percentage of expressed and non-expressed control gene pairs (CG2) among different TCGA datasets. X-axis, TCGA datasets. [file peerj-07-7107-s002.pdf]

Figure S3

A

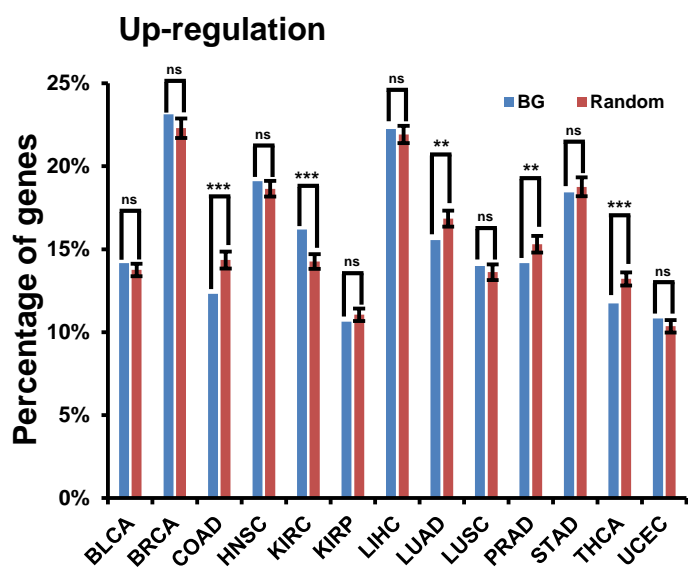

B

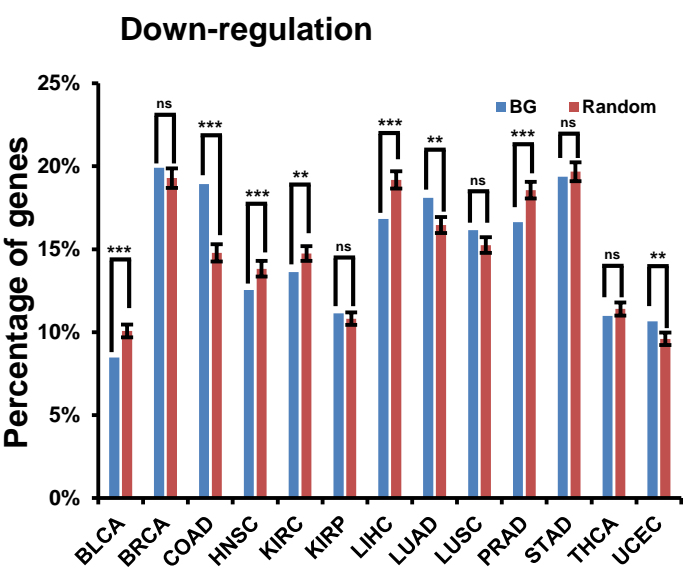

C

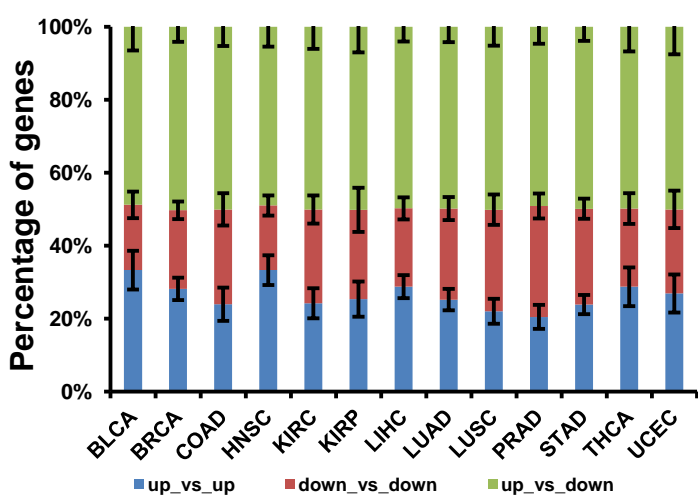

D

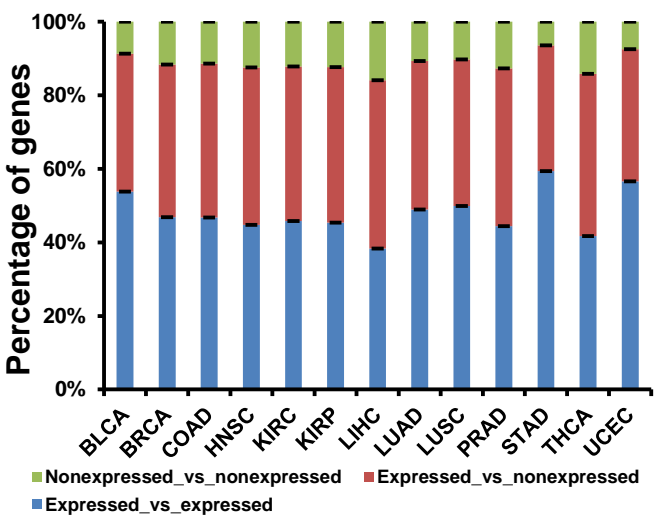

Supplement: Supplemental Information 3 — A. Percentage of up-regulated bidirectional and random genes among TCGA datasets. X-axis, TCGA datasets. The percentages in random genes represent mean of 100 random sets and the error bars represent the standard deviation. The p-values were computed by T test given the mean and standard deviation of random distribution. “ns” indicating not significant. “*” indicating p-value < 0.05. “**” indicating p-value < 0.01. “***” indicating p-value < 0.001. B. Percentage of down-regulated bidirectional and random genes among TCGA datasets. X-axis, TCGA datasets. The percentages in random genes represent mean of 100 random sets and the error bars represent the standard deviation. The p-values were computed by T test given the mean and standard deviation of random distribution. “ns” indicating not significant. “*” indicating p-value < 0.05. “**” indicating p-value < 0.01. “***” indicating p-value < 0.001. C. Percentage of the patterns of differentially expressed combination in random gene pair. X-axis, TCGA datasets. The percentages in random genes represent mean of 100 random sets and the error bars represent the standard deviation. The average number of specific combination is used to compute p-value in “chisq.test” function in R when comparing with bidirectional gene pairs. D. Percentage of expressed and non-expressed random gene pairs among different TCGA datasets. X-axis, TCGA datasets. The percentages in random genes represent mean of 100 random sets and the error bars represent the standard deviation. The average number of specific combination is used to compute p-value in “chisq.test” function in R when comparing with bidirectional gene pairs. [file peerj-07-7107-s003.pdf]

Figure S4

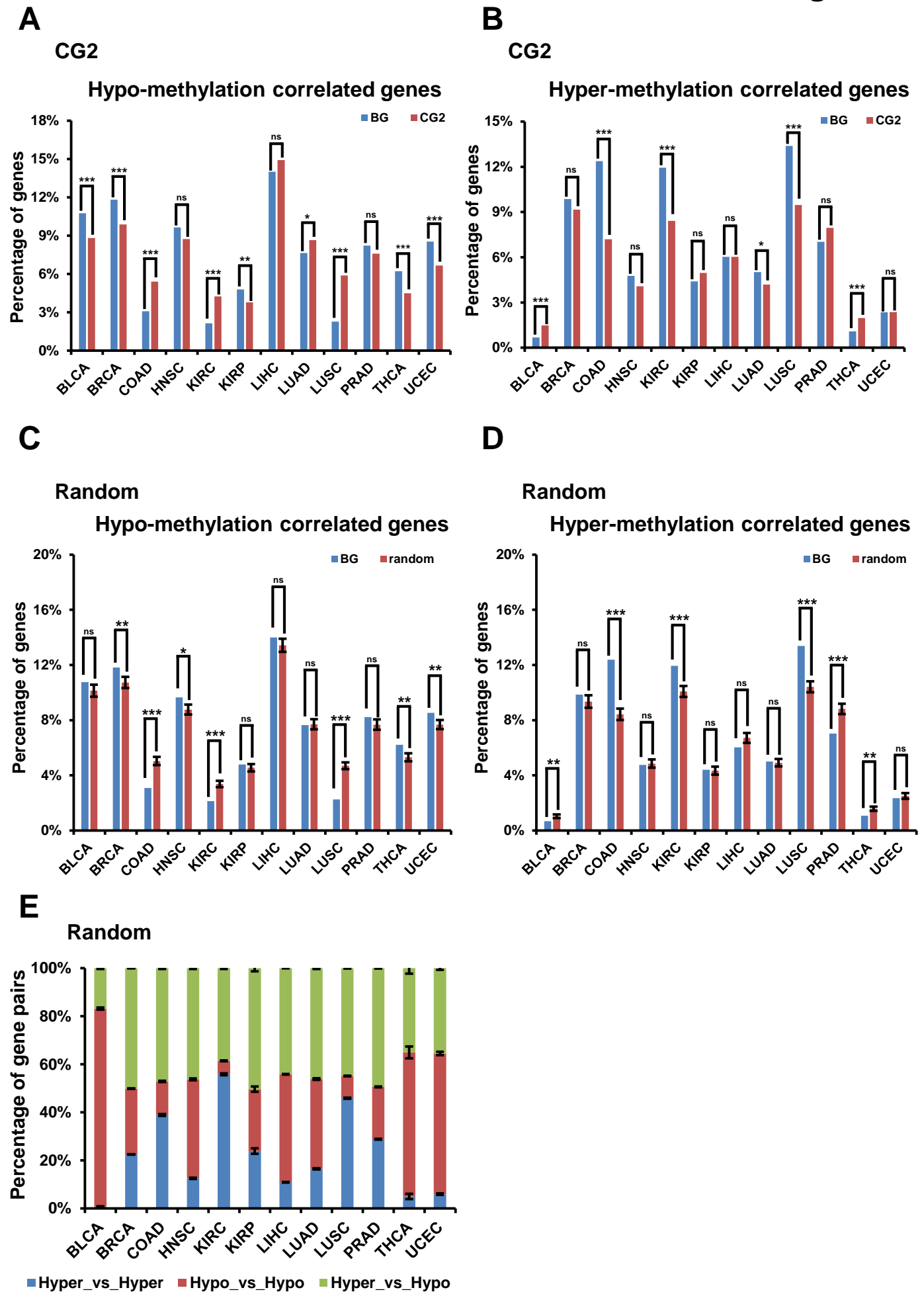

Supplement: Supplemental Information 4 — A. Percentage of hypo-methylation correlated bidirectional and control genes (CG2) in TCGA dataset. X-axis, TCGA datasets. The p-values were computed by “chisq.test” function in R. “ns” indicating not significant. “*” indicating p-value < 0.05. “**” indicating p-value < 0.01. “***” indicating p-value < 0.001. B. Percentage of hyper-methylation correlated bidirectional and control genes (CG2) in TCGA dataset. X-axis, TCGA datasets. The p-values were computed by “chisq.test” function in R. “ns” indicating not significant. “*” indicating p-value < 0.05. “**” indicating p-value < 0.01. “***” indicating p-value < 0.001. C. Percentage of hypo-methylation correlated bidirectional and random genes in TCGA dataset. X-axis, TCGA datasets. The percentages in random genes represent mean of 100 random sets and the error bars represent the standard deviation. The p-values were computed by T test given the mean and standard deviation of random distribution. “ns” indicating not significant. “*” indicating p-value < 0.05. “**” indicating p-value < 0.01. “***” indicating p-value < 0.001. D. Percentage of hyper-methylation correlated bidirectional and random genes in TCGA dataset. X-axis, TCGA datasets. The percentages in random genes represent mean of 100 random sets and the error bars represent the standard deviation. The p-values were computed by T test given the mean and standard deviation of random distribution. “ns” indicating not significant. “*” indicating p-value < 0.05. “**” indicating p-value < 0.01. “***” indicating p-value < 0.001. Percentage of combination of hypo- or hyper-methylation correlated random genes in TCGA dataset. X-axis, TCGA datasets. The percentages in random genes represent mean of 100 random sets and the error bars represent the standard deviation. The p-values were computed by “chisq.test” function in R. The number of specific combination in random gene pairs used in “chisq.test” is the mean of 100 random sets. [file peerj-07-7107-s004.pdf]
