# Supplementary material for: Characterization of bidirectional gene pairs in The Cancer Genome Atlas (TCGA) dataset
Source: PeerJ. 2019 Jun 17;7:e7107. doi: 10.7717/peerj.7107 (PMC6585903; doi:10.7717/peerj.7107)
Supplement: Supplemental Information 18 [file peerj-07-7107-s018.pdf]

# Table S1

**Table S1. Summary of the number of cancer and normal samples in each of the 13 analyzed TCGA datasets.**

| Cancer type | | Number of RNA-seq libraries analyzed | | | Number of methylation libraries analyzed | | | Number of patients with survival data | | |
|---|---|---|---|---|---|---|---|---|---|---|
| Abbreviation | Full name | Cancer | Healthy | Combined | Cancer | Healthy | Combined | Alive | Dead | Combined |
| BLCA | Bladder Urothelial Carcinoma | 414 | 19 | 433 | 418 | 21 | 439 | 231 | 181 | 412 |
| BRCA | Breast invasive carcinoma | 1102 | 113 | 1215 | 793 | 97 | 890 | 946 | 151 | 1097 |
| COAD | Colon adenocarcinoma | 478 | 41 | 519 | 313 | 38 | 351 | 357 | 102 | 459 |
| HNSC | Head and Neck squamous cell carcinoma | 500 | 44 | 544 | 528 | 50 | 578 | 305 | 223 | 528 |
| KIRC | Kidney renal clear cell carcinoma | 538 | 72 | 610 | 324 | 160 | 484 | 360 | 177 | 537 |
| KIRP | Kidney renal papillary cell carcinoma | 288 | 32 | 320 | 275 | 45 | 320 | 247 | 44 | 291 |
| LIHC | Liver hepatocellular carcinoma | 371 | 50 | 421 | 377 | 50 | 427 | 245 | 132 | 377 |
| LUAD | Lung adenocarcinoma | 533 | 59 | 592 | 473 | 32 | 505 | 334 | 188 | 522 |
| LUSC | Lung squamous cell carcinoma | 502 | 49 | 551 | 370 | 42 | 412 | 285 | 219 | 504 |
| PRAD | Prostate adenocarcinoma | 498 | 52 | 550 | 502 | 50 | 552 | 490 | 10 | 500 |
| STAD | Stomach adenocarcinoma | 375 | 32 | 407 | 395 | 2 | 397 | 271 | 172 | 443 |
| THCA | Thyroid carcinoma | 502 | 58 | 560 | 507 | 56 | 563 | 491 | 16 | 507 |
| UCEC | Uterine Corpus Endometrial Carcinoma | 551 | 35 | 586 | 438 | 46 | 484 | 457 | 91 | 548 |
