# Supplementary material for: Characterization of bidirectional gene pairs in The Cancer Genome Atlas (TCGA) dataset
Source: PeerJ. 2019 Jun 17;7:e7107. doi: 10.7717/peerj.7107 (PMC6585903; doi:10.7717/peerj.7107)
Supplement: Supplemental Information 19 [file peerj-07-7107-s019.pdf]

Table S2

Table S2. Summary of the number of prognostic protein coding genes in each of the 13 analyzed TCGA datasets.

Dataset	OS								DFI								PFI							
	BG		CG1		CG2		p-value		BG		CG1		CG2		p-value		BG		CG1		CG2		p-value	
	PG	NPG	PG	NPG	PG	NPG	BG.vs.CG1	BG.vs.CG2	PG	NPG	PG	NPG	PG	NPG	BG.vs.CG1	BG.vs.CG2	PG	NPG	PG	NPG	PG	NPG	BG.vs.CG1	BG.vs.CG2
BLCA	928	3,404	239	864	1,147	4,013	8.91E-01	3.56E-01	400	3,932	84	1,019	464	4,696	1.04E-01	7.10E-01	1,013	3,319	227	876	1,056	4,104	5.23E-02	6.59E-04
BRCA	579	3,699	143	942	650	4,307	7.98E-01	5.73E-01	478	3,800	147	938	610	4,347	3.36E-02	9.88E-02	451	3,827	142	943	605	4,352	1.96E-02	1.35E-02
COAD	490	3,773	129	957	586	4,395	7.64E-01	7.10E-01	254	4,009	98	988	360	4,621	3.58E-04	1.64E-02	587	3,676	163	923	739	4,242	3.17E-01	1.53E-01
HNSC	752	3,530	154	932	780	4,249	9.01E-03	8.45E-03	152	4,130	68	1,018	295	4,734	8.14E-05	2.45E-07	695	3,587	146	940	709	4,320	2.71E-02	4.56E-03
KIRC	2,193	2,055	546	529	2,345	2,564	6.50E-01	2.54E-04	240	4,008	64	1,011	302	4,607	7.57E-01	3.31E-01	2,077	2,171	495	580	2,234	2,675	1.02E-01	1.30E-03
KIRP	1,328	2,912	303	772	1,413	3,496	5.08E-02	8.83E-03	854	3,386	220	855	1,001	3,908	8.47E-01	7.87E-01	1,380	2,860	320	755	1,494	3,415	8.75E-02	3.16E-02
LIHC	884	3,284	165	886	870	3,889	8.14E-05	5.67E-04	686	3,482	136	915	651	4,108	5.94E-03	2.71E-04	757	3,411	133	918	714	4,045	2.71E-05	6.74E-05
LUAD	941	3,347	236	851	1,027	3,965	9.00E-01	1.13E-01	223	4,065	52	1,035	306	4,686	6.31E-01	6.01E-02	601	3,687	131	956	633	4,359	1.02E-01	6.31E-02
LUSC	218	4,092	72	1,026	358	4,711	5.82E-02	6.71E-05	327	3,983	75	1,023	400	4,669	4.30E-01	6.10E-01	572	3,738	148	950	652	4,417	8.96E-01	5.79E-01
PRAD	365	3,899	89	995	425	4,465	7.58E-01	8.53E-01	829	3,435	211	873	859	4,031	1.00E+00	2.26E-02	1,132	3,132	283	801	1,256	3,634	7.99E-01	3.61E-01
STAD	365	3,976	93	1,011	508	4,714	1.00E+00	2.81E-02	983	3,358	211	893	968	4,254	1.27E-02	7.92E-07	900	3,441	203	901	923	4,299	9.12E-02	1.68E-04
THCA	432	3,783	110	957	527	4,266	9.99E-01	2.66E-01	373	3,842	89	978	422	4,371	6.42E-01	9.70E-01	456	3,759	121	946	524	4,269	6.65E-01	8.89E-01
UCEC	897	3,450	236	869	1,080	4,147	6.26E-01	9.94E-01	615	3,732	141	964	677	4,550	2.53E-01	9.40E-02	1,193	3,154	266	839	1,233	3,994	2.62E-02	1.75E-05

PG: prognostic gene
NPG: non-prognostic gene
