# Supplementary material for: Characterization of bidirectional gene pairs in The Cancer Genome Atlas (TCGA) dataset
Source: PeerJ. 2019 Jun 17;7:e7107. doi: 10.7717/peerj.7107 (PMC6585903; doi:10.7717/peerj.7107)
Supplement: Supplemental Information 20 [file peerj-07-7107-s020.pdf]

Table S3

Table S3. Summary of the number of prognostic genes in each of the 13 analyzed TCGA datasets.

Dataset	OS								DFI								PFI							
	BG		CG1		CG2		p-value		BG		CG1		CG2		p-value		BG		CG1		CG2		p-value	
	PG	NPG	PG	NPG	PG	NPG	BG.vs.CG1	BG.vs.CG2	PG	NPG	PG	NPG	PG	NPG	BG.vs.CG1	BG.vs.CG2	PG	NPG	PG	NPG	PG	NPG	BG.vs.CG1	BG.vs.CG2
BLCA	1,326	5,111	324	1,186	1,842	7,127	4.81E-01	9.41E-01	718	5,719	155	1,355	1,091	7,878	3.43E-01	5.81E-02	1,332	5,105	319	1,191	1,787	7,182	7.35E-01	2.50E-01
BRCA	752	5,443	173	1,249	942	7,152	1.00E+00	3.73E-01	669	5,526	179	1,243	869	7,225	5.91E-02	9.26E-01	632	5,563	178	1,244	910	7,184	1.22E-02	4.99E-02
COAD	659	5,461	151	1,271	821	7,471	9.08E-01	9.56E-02	369	5,751	120	1,302	533	7,759	1.10E-03	3.47E-01	736	5,384	192	1,230	983	7,309	1.38E-01	7.74E-01
HNSC	933	5,115	202	1,179	1,193	6,692	4.82E-01	6.46E-01	199	5,849	73	1,308	414	7,471	4.95E-04	2.86E-08	853	5,195	173	1,208	976	6,909	1.36E-01	3.03E-03
KIRC	2,933	3,161	680	729	3,448	4,479	9.52E-01	5.24E-08	315	5,779	77	1,332	490	7,437	7.01E-01	1.18E-02	2,706	3,388	589	820	3,103	4,824	8.12E-02	4.11E-10
KIRP	1,758	4,386	378	1,032	2,036	5,872	1.85E-01	1.58E-04	1,088	5,056	266	1,144	1,432	6,476	3.26E-01	5.55E-01	1,781	4,363	395	1,015	2,111	5,797	4.87E-01	2.75E-03
LIHC	1,082	4,688	194	1,118	1,161	5,842	8.57E-04	1.42E-03	895	4,875	167	1,145	966	6,037	1.22E-02	6.68E-03	972	4,798	166	1,146	1,041	5,962	2.23E-04	2.42E-03
LUAD	1,178	5,044	282	1,165	1,411	7,010	6.54E-01	6.95E-04	325	5,897	75	1,372	512	7,909	1.00E+00	2.99E-02	804	5,418	167	1,280	979	7,442	1.68E-01	1.90E-02
LUSC	304	5,990	92	1,357	557	7,972	2.14E-02	1.43E-05	420	5,874	92	1,357	549	7,980	6.98E-01	5.88E-01	763	5,531	179	1,270	916	7,613	8.43E-01	9.33E-03
PRAD	508	5,633	120	1,289	695	7,078	8.06E-01	1.73E-01	1,120	5,021	266	1,143	1,315	6,458	6.02E-01	4.41E-02	1,526	4,615	358	1,051	1,924	5,849	6.87E-01	9.11E-01
STAD	500	6,085	119	1,446	783	9,088	1.00E+00	4.44E-01	1,326	5,259	276	1,289	1,639	8,232	2.76E-02	8.62E-09	1,166	5,419	259	1,306	1,445	8,426	2.95E-01	1.47E-07
THCA	656	5,335	143	1,226	847	6,684	6.22E-01	6.04E-01	520	5,471	108	1,261	620	6,911	3.73E-01	3.69E-01	652	5,339	153	1,216	795	6,736	7.91E-01	5.60E-01
UCEC	1,281	5,262	305	1,235	1,683	7,615	8.68E-01	2.00E-02	898	5,645	200	1,340	1,292	8,006	4.72E-01	7.77E-01	1,669	4,874	358	1,182	1,945	7,353	7.04E-02	1.39E-11

PG: prognostic gene
NPG: non-prognostic gene
