# Supplementary material for: Characterization of bidirectional gene pairs in The Cancer Genome Atlas (TCGA) dataset
Source: PeerJ. 2019 Jun 17;7:e7107. doi: 10.7717/peerj.7107 (PMC6585903; doi:10.7717/peerj.7107)
Supplement: Supplemental Information 21 [file peerj-07-7107-s021.pdf]

Table S4

Table S4. Summary of the number of prognostic protein coding gene pairs in each of the 13 analyzed TCGA datasets.

Dataset	OS								DFI								PFI							
	BG		CG1		CG2		p-value		BG		CG1		CG2		p-value		BG		CG1		CG2		p-value	
	PGP	NPGP	PGP	NPGP	PGP	NPGP	BG.vs.CG1	BG.vs.CG2	PGP	NPGP	PGP	NPGP	PGP	NPGP	BG.vs.CG1	BG.vs.CG2	PGP	NPGP	PGP	NPGP	PGP	NPGP	BG.vs.CG1	BG.vs.CG2
BLCA	342	904	142	279	556	1,231	1.67E-02	3.27E-02	113	1,133	40	381	207	1,580	8.67E-01	3.09E-02	363	883	115	306	484	1,303	5.15E-01	2.32E-01
BRCA	207	1,018	75	332	275	1,415	5.28E-01	6.90E-01	153	1,072	72	335	272	1,418	1.07E-02	7.61E-03	149	1,076	80	327	272	1,418	2.26E-04	3.42E-03
COAD	149	1,072	62	352	272	1,431	1.71E-01	4.97E-03	109	1,112	48	366	143	1,560	1.35E-01	6.62E-01	171	1,050	93	321	340	1,363	7.34E-05	3.54E-05
HNSC	280	946	85	329	348	1,377	3.64E-01	8.97E-02	52	1,174	32	382	141	1,584	7.94E-03	2.88E-05	282	944	72	342	297	1,428	1.98E-02	1.17E-04
KIRC	871	337	276	124	1,082	583	2.60E-01	6.44E-05	72	1,136	18	382	108	1,557	3.29E-01	6.19E-01	842	366	270	130	1,061	604	4.45E-01	9.50E-04
KIRP	588	621	171	232	688	976	3.55E-02	1.21E-04	382	827	120	283	520	1,144	5.35E-01	8.75E-01	617	592	173	230	742	922	5.75E-03	7.33E-04
LIHC	390	797	99	291	391	1,181	6.84E-03	4.97E-06	276	911	74	316	279	1,293	9.04E-02	4.27E-04	313	874	80	310	306	1,266	2.43E-02	2.07E-05
LUAD	360	866	122	287	473	1,237	9.08E-01	3.33E-01	50	1,176	24	385	125	1,585	1.71E-01	3.59E-04	170	1,056	62	347	263	1,447	5.71E-01	2.77E-01
LUSC	64	1,171	32	385	116	1,633	7.85E-02	1.19E-01	97	1,138	37	380	163	1,586	5.79E-01	1.83E-01	222	1,013	86	331	314	1,435	2.59E-01	1.00E+00
PRAD	166	1,053	49	361	210	1,452	4.37E-01	4.73E-01	312	907	105	305	379	1,283	1.00E+00	9.12E-02	512	707	155	255	635	1,027	1.51E-01	4.37E-02
STAD	109	1,129	38	383	211	1,614	9.69E-01	1.69E-02	288	950	86	335	411	1,414	2.56E-01	6.62E-01	315	923	91	330	413	1,412	1.30E-01	7.97E-02
THCA	168	1,029	48	352	238	1,364	3.44E-01	5.78E-01	149	1,048	47	353	154	1,448	7.79E-01	2.00E-02	162	1,035	68	332	218	1,384	1.04E-01	9.99E-01
UCEC	365	889	118	304	488	1,344	6.99E-01	1.43E-01	207	1,047	57	365	294	1,538	1.66E-01	7.72E-01	464	790	135	287	579	1,253	7.20E-02	2.11E-03

PGP: prognostic gene pair
NPGP: non-prognostic gene pair
