# Supplementary material for: Characterization of bidirectional gene pairs in The Cancer Genome Atlas (TCGA) dataset
Source: PeerJ. 2019 Jun 17;7:e7107. doi: 10.7717/peerj.7107 (PMC6585903; doi:10.7717/peerj.7107)
Supplement: Supplemental Information 22 [file peerj-07-7107-s022.pdf]

Table S5

Table S5. Summary of the number of prognostic gene pairs in each of the 13 analyzed TCGA datasets.

Dataset	OS								DFI								PFI							
	BG		CG1		CG2		p-value		BG		CG1		CG2		p-value		BG		CG1		CG2		p-value	
	PGP	NPGP	PGP	NPGP	PGP	NPGP	BG.vs.CG1	BG.vs.CG2	PGP	NPGP	PGP	NPGP	PGP	NPGP	BG.vs.CG1	BG.vs.CG2	PGP	NPGP	PGP	NPGP	PGP	NPGP	BG.vs.CG1	BG.vs.CG2
BLCA	813	2,191	210	455	1,276	3,086	2.14E-02	4.32E-02	423	2,581	89	576	650	3,712	6.83E-01	3.44E-01	836	2,168	185	480	1,160	3,202	1.00E+00	2.52E-01
BRCA	447	2,396	100	507	557	3,220	6.90E-01	2.89E-01	371	2,472	99	508	521	3,256	3.94E-02	4.00E-01	341	2,502	106	501	554	3,223	3.50E-04	1.86E-03
COAD	356	2,407	85	526	493	3,435	5.38E-01	7.14E-01	222	2,541	62	549	264	3,664	1.05E-01	4.65E-02	355	2,408	117	494	604	3,324	6.37E-05	4.10E-03
HNSC	551	2,157	118	464	694	2,847	1.00E+00	4.83E-01	95	2,613	39	543	259	3,282	6.26E-04	1.61E-10	514	2,194	86	496	517	3,024	2.01E-02	4.46E-06
KIRC	1,884	879	402	202	2,264	1,379	4.66E-01	6.19E-07	130	2,633	28	576	190	3,453	1.00E+00	3.84E-01	1,749	1,014	386	218	2,068	1,575	8.15E-01	1.50E-07
KIRP	1,168	1,654	230	376	1,319	2,317	1.30E-01	3.15E-05	713	2,109	159	447	948	2,688	6.55E-01	4.80E-01	1,177	1,645	235	371	1,376	2,260	1.99E-01	1.78E-03
LIHC	658	1,869	119	424	664	2,333	4.11E-02	8.43E-04	488	2,039	98	445	502	2,495	5.36E-01	1.48E-02	581	1,946	102	441	556	2,441	3.74E-02	5.52E-05
LUAD	735	2,109	159	463	865	3,126	9.25E-01	6.77E-05	143	2,701	30	592	285	3,706	9.12E-01	4.60E-04	441	2,403	86	536	538	3,453	3.20E-01	2.02E-02
LUSC	149	2,750	48	578	254	3,779	1.63E-02	4.76E-02	204	2,695	52	574	311	3,722	3.05E-01	3.13E-01	465	2,434	112	514	575	3,458	2.82E-01	4.38E-02
PRAD	323	2,489	67	538	468	3,057	8.27E-01	3.54E-02	698	2,114	149	456	776	2,749	9.61E-01	9.36E-03	1,026	1,786	219	386	1,246	2,279	9.31E-01	3.61E-01
STAD	251	2,829	59	637	423	4,594	8.35E-01	6.86E-01	758	2,322	124	572	993	4,024	1.59E-04	3.69E-07	652	2,428	140	556	904	4,113	5.72E-01	5.33E-04
THCA	389	2,313	83	498	468	2,918	9.97E-01	5.46E-01	311	2,391	62	519	337	3,049	6.13E-01	5.54E-02	376	2,326	91	490	446	2,940	3.04E-01	4.20E-01
UCEC	775	2,304	173	502	1,053	3,512	8.42E-01	3.68E-02	496	2,583	92	583	721	3,844	1.22E-01	7.36E-01	1,015	2,064	195	480	1,232	3,333	4.48E-02	2.14E-08

PGP: prognostic gene pair
NPGP: non-prognostic gene pair
